# ﻿Contributions to the world fauna of Microgastrinae parasitoid wasps (Hymenoptera, Braconidae) – Introduction

**DOI:** 10.3897/zookeys.1175.108529

**Published:** 2023-08-16

**Authors:** Jose L. Fernandez-Triana

**Affiliations:** 1 Canadian National Collection of Insects, Arachnids and Nematodes, Ottawa, Canada Canadian National Collection of Insects Ottawa Canada

Microgastrinae (Hymenoptera: Braconidae) is a hyper-diverse group of insects, with more than 3,100 described species and estimates of up to 50,000 species worldwide (Rodriguez et al. 2013, Fernandez-Triana et al. 2020). Because they are exclusively parasitoids of larval Lepidoptera, many species have been used in or considered for biological control projects of agricultural and forestry pests (Whitfield 1997), and the group has been studied in the context of many areas of ecological, agricultural, and basic science (Whitfield et al. 2018).

The pace of species description in Microgastrinae has been steadily increasing since the first species was described in 1758 and has shown no signs of slowing down. From 2014 to 2019 a total of 720 new species was described, an average of 120 new species/year which represented the largest increase for any subfamily of Braconidae in that time span (Yu et al. 2016; Fernandez-Triana et al. 2020). That means almost 1% of the estimated 18,000 new species that are described every year, a remarkable feat considering microgastrines only represent a small fraction of the parasitoid wasp diversity worldwide.

During the past 15 years ZooKeys has played a significant role in the advancement of Microgastrinae research, with at least 36 papers being published in the journal between 2009 and 2023 (https://zookeys.pensoft.net/browse_journal_articles?&search_hidden=microgastrinae&search_in=0&sortby=6). The majority were taxonomic revisions, but some also covered other topics such as checklists and faunistics, and at least four papers primarily dealing with Lepidoptera also recorded new host-parasitoid associations that included microgastrine wasps. Altogether, those papers covered all biogeographical regions, describing 407 new species and three new genera, and proposing more than 350 additional nomenclatural acts (new combinations, lectotype designations, revised status, etc.).

Despite that progress, more than 90% of the world species remain undescribed, mostly in tropical areas (Fig. 1); even in temperate areas (Holarctic) it is here estimated that at most one quarter of all species are known. Beyond species richness, much less is known about parasitoid biology and ecology, accurate host ranges, species distribution, DNA data, etc.

**Figure 1.**
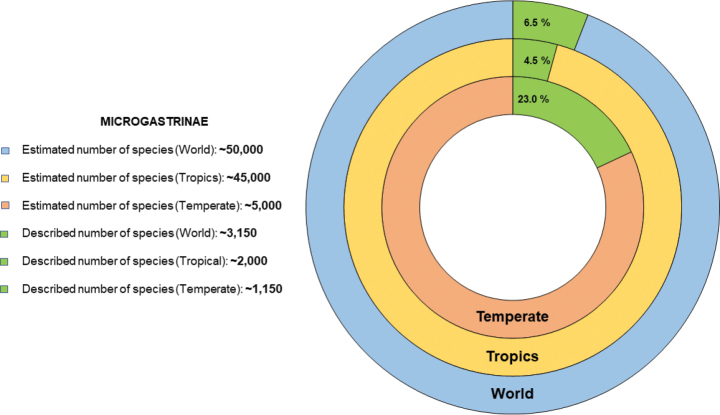
Described and estimated species richness of the Microgastrinae fauna, at world, tropical, or temperate level. Data on described species based on Fernandez-Triana et al. (2020), updated to include references published after 2019; estimated species richness based on the highest values reported by Rodriguez et al. (2013) and Fernandez-Triana et al. (2020), and unpublished data from the author. All figures are rounded

A recent ZooKeys monograph (Fernandez-Triana et al. 2020), listing all world species of Microgastrinae, has provided a foundation for future studies that will hopefully further increase our understanding of this important group of parasitoid wasps. Thus, this collection of articles will be devoted to comprehensive taxonomic revisions and/or compilations of regional checklists, with an emphasis on turbo taxonomy approaches to speed up species description, including extensive use of DNA barcoding, and cataloguing of species for specific regions of the planet.

The first paper of the collection is a comprehensive revision of the world fauna of *Alphomelon* (a genus widely distributed in the New World), but it is hoped that it will be followed by a variety of papers dealing with the faunas of Australia, Botswana, Canada, Costa Rica, French Guiana, Germany, India, Israel, Japan, Madagascar, New Zealand, Scandinavia, Thailand, Uganda, and the United States, among other areas. These upcoming papers are a testament to the efforts of the international community of braconid researchers working with this fascinating, important, and still poorly understood group of parasitoid wasps.

## ﻿Additional information

### Author ORCIDs

Jose Fernandez-Triana https://orcid.org/0000-0003-0425-0309

### Data availability

All of the data that support the findings of this study are available in the main text.
